# Supplementary material for: Supplementation of Probiotic Butyricicoccus pullicaecorum Mediates Anticancer Effect on Bladder Urothelial Cells by Regulating Butyrate-Responsive Molecular Signatures
Source: Diagnostics (Basel). 2021 Dec 4;11(12):2270. doi: 10.3390/diagnostics11122270 (PMC8700285; doi:10.3390/diagnostics11122270)
Supplement: Supplementary file 1 [file diagnostics-11-02270-s001.zip › diagnostics-1419260-supplementary.pdf]

Supplemental Figures

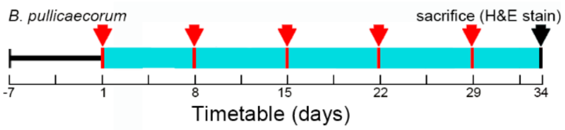

Supplemental Figure S1. Timing of *B. pullicaecorum* administration. Red arrow, 1 × 10^7 *B. pullicaecorum* administration. Black arrow, sacrifice and colon sampling of H&E staining.

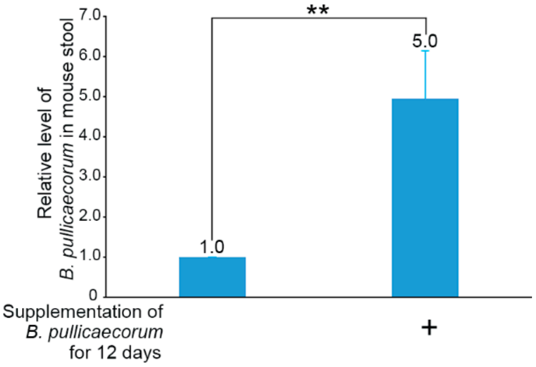

Supplemental Figure S2. Relative level of *B. pullicaecorum* in mouse stool. DNA was extracted from stools of mice without or with *B. pullicaecorum* supplementation for 12 days. The quantitative real-time PCR was performed with the Microbial DNA qPCR Assays (BP00080A; Qiagen GmbH) containing one pair of PCR primers and probe for *B. pullicaecorum*, according to the manufacturer's instructions. Each quantitation was normalized with the individual total bacteria level which was acquired by applying the SYBR green method and the universal bacterial primers (5'-GTGSTGCAYGGYYGTCGTCA-3' and 5'-ACGTCRTCCMCNCCTTCCTC-3'; S, C/G; Y, C/T; R, A/G; M, A/C; N, A/C/G/T), published by Walker *et al*. (Microbiome 2015; 3:26). Each experiment was repeated three times independently. **$p < 0.01$.

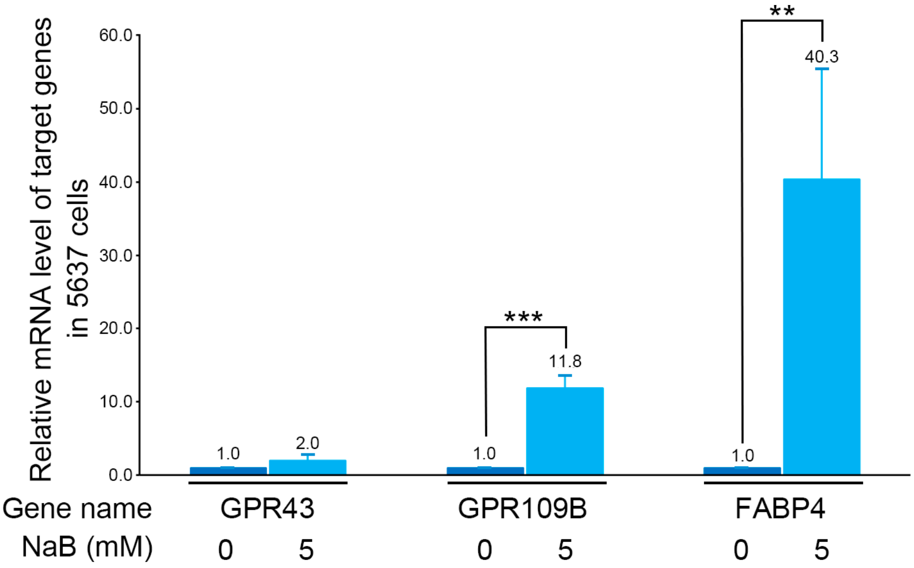

Supplemental Figure S3. Relative mRNA level of butyrate-effected genes in 5637 cells. Expressions of GPR43, GPR109B, and FABP4 were determined by quantitative real-time PCR from 5637 cells with 5 mM treatment for 72 h. The concentration of NaB was referred to the report of Wang *et al*. (FASEB J. 2020 34(3):4266). Each experiment was repeated three times independently. NaB, sodium butyrate. **$p <$

0.01; ***$p < 0.001$.

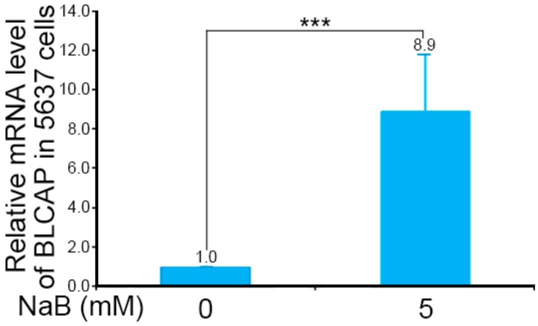

Supplemental Figure S4. Relative mRNA level of BLCAP in 5637 cells. Expressions of BLCAP were determined by quantitative real-time PCR from 5637 cells with 5 mM treatment for 72 h. The concentration of NaB was referred to the report of Wang *et al*. (FASEB J. 2020 34(3):4266). Each experiment was repeated three times independently. NaB, sodium butyrate. ***$p < 0.001$.
